# Foundations of attention sharing: Orienting and responding to attention in term and preterm 5-month-old infants

**DOI:** 10.1016/j.infbeh.2020.101466

**Published:** 2020-11

**Authors:** Merideth Gattis, Alice Winstanley, Rebecca Sperotto, Diane L. Putnick, Marc H. Bornstein

**Affiliations:** a School of Psychology, Cardiff University, UK; b Eunice Kennedy Shriver National Institute of Child Health and Human Development, National Institutes of Health, Bethesda, MD, USA; c Institute for Fiscal Studies, London, UK; d UNICEF, New York, NY, USA

**Keywords:** Attention, Contingency, Infancy, Parenting, Prematurity, Responsiveness

## Abstract

Attention is the gateway to perceptual, cognitive, and socioemotional development in humans. We observed 104 5-month-old term and preterm infants and their mothers in social interactions to address three questions about the role of maturation in orienting and responding to attention. We used a fine-grained coding system to allow parallel comparisons across infant and maternal orienting, and sequential analysis to evaluate infant and maternal responding to attention. Orienting and responding to attention differed for attention to people versus objects, as did the relations between maturity and attention. We conclude that maturity contributes to orienting and responding to attention and that orienting and responding to attention are specific rather than homogenous. We discuss the implications of these conclusions for future studies of how attention influences cognitive and communicative development.

## Highlights

• Investigated attention in full-term and preterm 5-month-olds and their mothers.
• Compared attention to persons and objects.
• Evaluated role of maturity in orienting and responding to attention.
• Infant attention to persons versus objects was related to maturity.
• Infant and maternal responsiveness were related to maturity.

## 1 Introduction

Attention determines where to look and who or what to respond to in the environment. In consequence, attention is a first-order psychological variable. Attention changes across the first year of life, and increasingly allows infants to selectively orient and share attention with social partners, which in turn supports cognitive, communicative, and socioemotional development (Bornstein, Putnick, Cote, Haynes, & Suwalsky, 2015; Bornstein, Arterberry, & Mash, 2015; Colombo, 2001; Mundy & Newell, 2007; Ristic & Enns, 2015). In the current study, we compared attentional processes in 5-month-old infants born term and preterm to evaluate the relations between maturity and orienting and responding to attention. In the following sections we first briefly review the literature on the development of attention in infancy, including three factors that influence attention sharing: how infants distribute their attention between people and objects, how infants respond to the attention of others, and how parents respond to their infants’ attention. We then review what is known about the development of orienting and responding to attention in preterm infants and their parents, and on this basis introduce our study.

### 1.1 Orienting attention to people and objects

Visual attention is immature at birth and matures dramatically across the first year of life (Atkinson, 2000; Colombo, 2001; Johnson, 1990; Mundy & Newell, 2007; Mundy, 2018). One of the most important changes in visual attention during the first year is in the control of attention, which shifts from limited, obligatory attention in the first weeks of life, to controlled orienting and sustaining of attention (Colaizzi, Aubuchon-Endsley, Grant, Kennedy, & Thomas, 2014; Ruff & Rothbart, 2001). Both behavioral and physiological data indicate that orienting attention increases between 3 and 6 months (Colaizzi et al., 2014; Colombo, 2001). By about 6 months, the processes involved in orienting attention are established, although maturation of attention continues throughout infancy and childhood (Colombo, 2001; Tsurumi, Kanazawa, & Yamaguchi, 2018).

The distribution of attention also changes during infancy, from primarily attending to people in the first weeks of life to primarily attending to objects by about the mid-point of the first year (Bornstein, Cote, & Kwak, 2019; Brazelton, Koslowski, & Martin, 1974; Johnson, Dzierawiec, Ellis, & Morton, 1991; Kaye & Fogel, 1980; Peltola, Yrttiaho, & Leppänen, 2017). Evaluating infant attention to people versus objects has the potential to yield insights relevant to both typical and atypical development: attention to both people and objects is necessary for sharing attention with a social partner, which in turn plays an important role in cognition and communication (Klin, Shultz, & Jones, 2015; Mundy & Newell, 2007; Mundy, 2018; Niedzwiecka, Ramotowska, & Tomalski, 2018; Peltola et al., 2017; Salley & Colombo, 2016; Salley et al., 2016). In one study evaluating this distribution of infant attention, parents played with their term infant at 2, 3, and 4 months in laboratory-based sessions (Perra & Gattis, 2012). At 2 months, infant attention was unfocused for the majority of the session, but nonetheless most infants demonstrated person engagement by orienting attention to parents at some point during the observation. In contrast, no infants demonstrated object engagement at 2 months, and most infants demonstrated object engagement for the first time at 4 months. In addition, Perra and Gattis (2012) reported that person engagement remained at similar levels across the 2, 3, and 4-month longitudinal sessions, but object engagement increased significantly from 3 to 4 months. However, the coding system used by Perra and Gattis was not designed to contrast the distributions of attention to people versus objects, and other behavioral categories also included some aspects of attention to objects. For example, passive joint engagement, a behavioral category in which infants attended to an object held by a caregiver but did not overtly interact with it, had a later onset than person engagement but an earlier onset than object engagement. In sum, the distributions of attention to people versus objects may be influenced by maturation, much like changes in orienting and sustaining attention, but further studies with more specific evidence are needed.

### 1.2 Responding to attention

As infants become capable of orienting and sustaining attention, they also respond to the attention of others, and as a result share attention with social partners. Infants begin responding to caregiver attempts to solicit their attention at around 2–3 months, and become increasingly skilled over the remaining year (Bakeman & Adamson, 1984; Mundy & Newell, 2007; Perra & Gattis, 2010, 2012; Tamis-LeMonda & Bornstein, 1990; Van Egeren, Barratt, & Roach, 2001). Attention sharing is not, however, entirely dependent on the infant’s capacity to respond to bids: Attention sharing can also arise from caregivers monitoring and following infant attention, which in turn supports infants in sustaining attention (Bakeman & Adamson, 1984; Landry, Chapieski, & Schmidt, 1986; Perra & Gattis, 2012; Suarez-Rivera, Smith, & Yu, 2019; Yu & Smith, 2016). Few studies have compared parent and infant responding to attention to evaluate their relative contributions to attention sharing. One study of parent interactions with 4-month-old infants found that parents were generally more responsive to infants than infants were to parents, and that parents followed infants’ activities and vocalizations more often than infants followed parents’ activities and vocalizations (Van Egeren et al., 2001). The exception to this pattern was responding to object-directed attention: 4-month-olds followed mothers’ object play more often than mothers followed infants’ object play.

Surprisingly little is known about the relation between orienting attention to people versus objects and responding to attention. Several researchers proposed that shifting attention from people to objects helps infants make the transition to responding to the attentional focus of their social partners (Bakeman & Adamson, 1984; Brazelton et al., 1974; Kaye & Fogel, 1980). In addition, studies of parental responding to infants provide some evidence that responding is not homogenous, but differs across domains of behavior (Bornstein & Tamis-LeMonda, 1990; Bornstein et al., 1992; Bornstein, Tamis-LeMonda, Hahn, & Haynes, 2008; Bornstein, Cote, Haynes, Suwalsky, & Bakeman, 2012; Van Egeren et al., 2001; Vibbert & Bornstein, 1989). Infant and parent responding to attention may therefore differ according to the focus of attention, in particular attention to people versus objects.

### 1.3 Orienting and responding to attention in preterm infants

Attention is important to development in children born preterm, but evidence about preterm orienting and responding is inconsistent (Boyd et al., 2013; Ross-Sheehy, Perone, Macek, & Eschman, 2017; van de Weijer-Bergsma, Wijnroks, & Jongmans, 2008). Preterm infants have been described as less engaged in social interactions compared to full-term infants, and some studies indicate that preterm infants and children look longer at visual stimuli and are slower at shifting attention to new visual targets (Brachfeld, Goldberg, & Sloman, 1980; Landry, Smith, Miller-Loncar, & Swank, 1997). In other experimental and observational studies, however, preterm infants have demonstrated similar levels of attention as term infants (Barratt, Roach, & Leavitt, 1992; Landry, 1986). In yet others, preterm infants have been reported to perform better than term infants, for example on attention orienting tasks (Butcher, Kalverboer, Geuze, & Stremmelaar, 2002; Hunnius, Geuze, Zweens, & Bos, 2008). In some cases, preterm infants have problems with attention that are due to comorbidities or medical complications rather than prematurity itself, but inconsistencies in evidence about preterm orienting and responding remain, even after accounting for (or in some studies, controlling for) comorbidities and medical complications (Downes, Kelly, Day, Marlow, & de Haan, 2018; Kavsek & Bornstein, 2008; Ross-Sheehy et al., 2017). To account for these inconsistencies, researchers have argued that when studies compare preterm and full-term infants based on conceptional or post-menstrual age, rather than chronological or birth age, preterm infants have an advantage due to greater extrauterine experience, particularly for aspects of early development that are dependent on visual experience (Atkinson, 2000; Butcher et al., 2002; Hunnius et al., 2008; Peña, Arias, & Dehaene-Lambertz, 2014; Ross-Sheehy et al., 2017).

The evidence about whether parental orienting and responding to infant attention is influenced by infants’ differential maturity is also inconsistent (Clark, Woodward, Horwood, & Moor, 2008; Gattis, 2019; Landry, Smith, Miller-Loncar, & Swank, 1997). Some studies have described parents of preterm infants as more active and attentive, responding to their infants’ attention and vocalizations more than parents of term infants (Barratt et al., 1992; Brachfeld et al., 1980). Other studies have reported that parents of preterm infants respond to their infants less frequently, direct their infants’ attention more frequently, and are more intrusive and controlling than parents of term infants (Forcada-Guex, Pierrehumbert, Borghini, Moessinger, & Muller-Nix, 2006; Garner, Landry, & Richardson, 1991; Loi et al., 2017). Few studies have examined infant and parental attention in a comparable manner across social partners, making it difficult to evaluate whether and how parental orienting and responding are influenced by infant attention and/or prematurity. To understand attention in infants born preterm and their parents, it is necessary to differentiate attention to people versus objects and examine whether the focus of attention differently affects orienting and responding. In other words, orienting and responding to attention need to be evaluated separately for attention to people versus objects.

### 1.4 This study

To meet this need, we conducted a study of orienting and responding to attention during interactions between 5-month-old term and preterm infants and their mothers. We examined orienting and responding using a fine-grained coding system to allow parallel comparisons and sequential analyses across infants and mothers (Bornstein et al., 2012; Bornstein, Putnick, Cote, Haynes, & Suwalsky, 2015; Bornstein & Tamis-LeMonda, 1990; Bornstein et al., 2008; Bornstein et al., 1992; Vibbert & Bornstein, 1989). We recruited a sample of healthy infants born between 30 and 42 weeks gestational age. Moderate to late preterm infants (born at 32–36 weeks gestation) are less well studied despite accounting for 84 % of preterm deliveries (Blencowe et al., 2012). We controlled for comorbidities and medical complications by carefully screening our sample. We controlled for extrauterine experience by observing all infants at the same chronological age (Brachfeld et al., 1980; Peña et al., 2014; Wilcox, Weinberg, & Basso, 2011). We examined maturity from 30–42 weeks gestational age as a continuous variable, rather than reducing maturity to a dichotomy of preterm versus term. We examined gestational age into the term period (37–42 weeks’ gestational age) given evidence that cognitive abilities vary with gestational age beyond term (Noble, Fifer, Rauh, Nomura, & Andrews, 2012; Yang, Platt, & Kramer, 2010).

The present study had three aims. First, we examined whether the distribution of attention between people and objects is affected by maturity. To accomplish this aim, we compared the distribution of attention to people and objects for healthy, low-risk preterm and term 5-month-olds and their mothers. Five months is a transitional period for orienting and responding to attention during which, based on previous experimental and observational evidence from term and preterm infants, it is possible to observe the influence of maturation (Colombo, 2001; Cote & Bornstein, 2018; Perra & Gattis, 2012; Rose, Feldman, & Jankowski, 2001; Ross-Sheehy et al., 2017; Tsurumi et al., 2018; Van Egeren et al., 2001). We tested all infants at the same chronological age to control for visual and social experience. If maturity influences the distribution of attention between people and objects, gestational age should be related to the allocation of attention to people versus objects.

Second, we tested the hypothesis that responding to attention is specific rather than homogenous across different foci of attention, to people versus to objects. Inconsistencies in previous findings about responding to attention in preterm infants and their parents might be due to a failure to distinguish between attention to people versus objects. Based on previous findings that parental responding differs between the two domains (e.g., Bornstein et al., 2012; Van Egeren et al., 2001), we considered that the relation between gestational age and infant and maternal responding to attention might therefore differ for people versus objects.

Third, we examined who leads and who follows in attention sharing when infants are 5 months, and whether leading versus following differs according to maturity. Based on Van Egeren et al.’s (2001) finding that caregivers generally follow infants’ activities and vocalizations more often than infants follow caregivers’ activities and vocalizations, we predicted that mothers would follow infants’ focus of attention more often than infants would follow mothers’ focus of attention. We noted, however, that Van Egeren et al. (2001) reported different patterns of following for different modalities of interaction (in their study, mothers’ object play versus mothers’ vocalizations). As with responding to attention, because of the diversity of previous findings about the effects of prematurity on infant and parent attention, we did not make a prediction about whether maturity affects leading versus following. Instead, much like our prediction that the relation between gestational age and infant and maternal responding to attention might differ for attention to people versus objects, we predicted that the relation between gestational age and leading versus following might differ across people and objects.

## 2 Method

### 2.1 Participants

A total of 104 dyads visited the university when infants were chronologically 5 months of age (+15 days based on chronological age) as part of a study about the development of preterm (30 ≤ GA≥36^+6^, *n* = 40) and term (37 ≤ GA≥42^+6^, *n* = 64) infants. Researchers recruited the majority of participants during the hospitalization period following delivery through the University Hospital Wales (*n* = 89) and recruited 15 parents through the Cardiff Birth Registry and other community links, such as the National Childbirth Trust. Mothers provided informed consent to participate in the study at recruitment. All study procedures were reviewed and approved by Cardiff and Southeast Wales Local Research Ethics Committee of the National Health Service of Wales (REC reference 10/WSE04/42, Protocol SPON 586-08, IRAS project ID 45603).

The primary inclusion criterion was gestational age: infants born between 30 and 42 weeks were eligible for the study. Exclusion criteria were: multiple births, mothers under 16 years old, and any serious medical conditions other than prematurity or any congenital abnormalities that could affect growth and development, including any surgical intervention during hospitalization. Three infants (two preterm and one term) were diagnosed with visual complications (or complications that could affect vision) between birth and the 5-month visit (one with retinopathy of prematurity, one with torticollis, and one with strabismus). In addition, one infant had a 5-min Apgar score of 5 (all other infants’ scores were 7 or higher). None of these four infants was an outlier on any outcome variable studied, and so all were included in all reported analyses.

Table 1 reports mother characteristics. Gestational age was not related to any characteristics of mothers in our sample: maternal age, parity, marital status, education, ethnicity, or family income. Table 2 reports infant characteristics. Infants born at younger gestational ages were also born at lower birthweights, spent more days in the hospital after birth, and had lower 5-minute Apgar scores.

**Table 1:** Mother characteristics.

			Preterm *N* = 40	Term *N* = 64	Total Sample *N* = 104	Association with gestational age
Age (years)	*M*(*SD*)		31.88 (4.97)	32.19 (4.41)	32.07 (4.62)	*r*(102) = −.01, *p* = .940
Parity	*N* (%)	First born	23 (58)	42 (66)	65 (63)	*t*(102) = 0.83, *p* = .408, *d* = 0.17
		Later born	17 (43)	22 (34)	39 (38)	
Marital status	*N* (%)	Single	7 (18)	9 (14)	16 (15)	*r*(102) = .07, *p* = .483
		Co-habiting	6 (15)	12 (19)	18 (17)	
		Married	27 (68)	43 (67)	70 (67)	
Maternal education	*N* (%)	GCSEs	5 (13)	6 (9)	11 (11)	*r*(101) = .13, *p* = .203
		A-Levels	7 (18)	7 (11)	14 (13)	
		Bachelor’s	13 (33)	25 (39)	38 (37)	
		Postgraduate	15 (38)	25 (39)	40 (39)	
Maternal ethnicity	*N* (%)	White	34 (94)	61 (97)	95 (96)	*t*(97) = 1.31, *p* = .195, *d* = 0.55
		Other	2 (6)	2 (3)	4 (4)	
Family income	*N* (%)	Less than £14,999	7 (18)	4 (6)	11 (11)	*r*(99) = .15, *p* = .128
		£15,000 - £39,999	11 (28)	16 (26)	27 (27)	
		Over £40,000	21 (54)	42 (68)	63 (62)	

*Note.* Data were missing for maternal education for 1 dyad, maternal ethnicity for 5 dyads, family income for 3 dyads.

**Table 2:** Infant characteristics.

			Preterm *N* = 40	Term *N* = 64	Total Sample *N* = 104	Association with gestational age
Gestational age (GA weeks)		*M* (*SD*)	34.41 (1.71)	39.91 (1.43)	37.79 (3.09)	
Chronological age (days)		*M (SD)*	152.25 (5.97)	153.22 (6.72)	152.98 (6.15)	*r*(102) = −.01, *p* = .953
Corrected age (days)		*M (SD)*	113.28 (10.89)	151.08 (14.83)	136.54 (22.82)	*r*(102) = .91, *p* <.001
Gender	Female	*N* (%)	16 (40)	29 (45)	45 (43)	*t*(102) = −0.83, *p* = .409, *d* = 0.16
	Male	*N* (%)	24 (60)	35 (55)	59 (57)	
Birthweight (grams)		*M* (*SD*)	2166.06 (473.85)	3436.85 (568.48)	2962.82 (807.58)	*r*(98) = .85, *p* <.001
Duration of hospitalization (days)		*Median* (*IQR*)	8.00 (20.50)	2.00 (2.00)	3.00 (4.50)	*ρ*(95) = −.75, *p* <.001
5-min Apgar		*Median* (*IQR*)	9.00 (1.00)	10.00 (1.00)	10.00 (1.00)	*ρ*(95) = .28, *p* = .005

*Note.* Data were missing for birthweight for 1 preterm infant and 2 term infants, duration of hospitalization for 2 preterm infants and 5 term infants, and 5-min Apgar scores for 2 preterm infants and 9 term infants. Duration of hospitalisation and 5-min Apgar scores were not normally distributed therefore descriptive statistics are reported as the median and interquartile range, and associations with gestational age were examined using Spearman’s rhos.

### 2.2 Procedure

Researchers scheduled observations at a time that the infant was normally awake and alert. The observations lasted 15 min. Three dyads terminated at a mean of 13 min due to infant tiredness; these dyads were not outliers on any dependent measure, so their data were prorated to 15 min.

The observation room contained a dark rectangular mat (130 cm by 190 cm), a soft cushion, an infant seat, and 3 toy bins containing a selection of 15 age-appropriate toys. A researcher asked mothers to play with the infant “as you would normally do at home.” The researcher told mothers that they could position their infant as they liked within the mat but asked them to remain on the mat to ensure that both members of the dyad would be within the range of the recording equipment.

### 2.3 Coding and analytic strategy

Researchers coded video-recorded interactions using mutually exclusive and exhaustive categories for infant and maternal person- and object-oriented attention (Bornstein, Cote, Haynes, Suwalsky, & Bakeman, 2012; Bornstein, Putnick, Cote, Haynes, & Suwalsky, 2015; Bornstein & Tamis-LeMonda, 1990; Bornstein, Tamis-LeMonda, Hahn, & Haynes, 2008; Bornstein et al., 1992; Vibbert & Bornstein, 1989) using Interact software (Mangold, 2009). The coding categories identified specific, corresponding attention behaviors for infants and mothers as events with onsets and offsets (Bakeman, Deckner, & Quera, 2005; Miller, 2011). In addition, to maximize accuracy and strengthen causal inferences, the independent event coding for attention behaviors for infants and mothers allowed for subsequent analysis of timed event sequences in the form of the likelihood of an appropriate response within a specific timeframe using sequential analysis (Bakeman & Quera, 2011). Birth status was stored separately from video-records to allow blind coding.

Infant attention was coded as: (a) looking at mother, (b) looking at an object, or (c) none of the above. The coding definition for looks required focused fixation, i.e. more than a fleeting glance, a blank stare, or a brief pass of the target during an episode of visual tracking. Other descriptive criteria for focused fixation included the brightening of the baby’s face or widening of the eyes when he/she first looked toward something, stilling all movement when the baby first focused on something, or moving in an excited way by waving arms or reaching toward the person or object. In addition to focused fixation, coded looks required the target of the fixation to be clear to the coder. A new event was coded when infants switched attention from one focus, whether within or between categories (i.e. from one object to another object or from object to person). For example, a new code started when the infant shifted attention from a book to a ball.

Maternal attention was coded as: (a) encouraging attention to herself, (b) encouraging attention to an object, or (c) none of the above. Maternal attention categories were thus similar to the infant attention categories in that they distinguished between person versus object attention and were defined with reference to how mothers attempted to guide infant attention. Maternal attempts to encourage attention could be physical (e.g., points to self, moves baby into a position to better look at her or an object, or shows the baby how to use an object) or verbal (e.g., “look at mommy”, naming the object/person or describing properties of the object/person). A new event was coded when mothers switched from one focus to another, whether within or between categories (i.e. from one object to another object or from object to person).

Frequencies and durations for each infant and mother attention code were tallied. Scores for duration were calculated as the proportion of the interaction. Scores for average duration (s) were calculated by dividing the total duration (s) of each code by the frequency of behavior.

Onsets and offsets of infant and mother attention were recorded to the nearest frame (or 0.04 s) to ensure timed event-sequential data for sequential analysis (Bakeman & Quera, 2011). Coders were first trained to reliability (Cohen, 1960, kappa: *κ* > 0.60) on a standard set of video-records. Coder reliability was checked regularly to protect against coding drift. For primary coding, the second author coded mother behavior and the third author coded infant behavior, thus allowing coding for the two social partners to be independent for sequential analysis. For reliability coding, the roles were reversed. Intercoder agreement was calculated as second-by-second agreement using approximately 20 % of interactions (maternal attention: mean *κ* = .66, mean percent agreement = 80 %; infant attention: mean *κ* = 0.58, mean percent agreement = 75 %). The timing requirements of the coding were stringent and may have lowered agreement, but the agreement and kappas both fall in acceptable ranges (Landis & Koch, 1977).

To evaluate infant and mother responding to attention, sequential analysis was used to create 4 sequential interaction variables that summarized behavioral streams following procedures described by Bakeman and Quera (2011). These variables assessed the odds of: (a) infant attention to mother given the mother was encouraging the infant to look at her; (b) mother encouraging attention to herself given the infant was looking at the mother; (c) infant looking to an object given the mother was encouraging attention to the same object; and (d) mother encouraging attention to an object given the infant was looking at the same object. A time window was set so that a response behavior had to occur within 3 s of the onset of the initial behavior (Bornstein et al., 2012; Van Egeren et al., 2001). For each dyad, time units were tallied in four 2 (given vs. not given) by 2 (target vs. not target) tables, one for each interactive variable, and an odds ratio (OR) was computed for each table. The OR is a descriptive measure of effect size (Bakeman et al., 2005). An OR < 1 indicates that the response behavior is more likely to occur within 3 s of the onset of the initial behavior than at other times, whereas a value between 0 and 1 reflects the response behavior was less likely to occur. Interact data files were converted to SDIS files using the ActSds software (Bakeman & Quera, 2008) so ORs could be computed using the Generalized Sequential Querier program (GSEQ version 5; Bakeman, Quera, & Gnisci, 2009).

For some dyads and interactive behaviors, we did not compute an OR due to insufficient data. The value of the OR was regarded as missing if the row total or column total for the contingency table of a dyad was five or fewer (Bakeman & Quera, 2011). Five percent of participants were missing data for infant responding and 9% for mother responding to person-oriented attention. Only 1 dyad was missing data for mother responding to infant object-oriented attention, and no dyads were missing data for infant responding to maternal object-oriented attention.

## 3 Results

### 3.1 Preliminary analyses

Prior to data analysis, all infant and mother attention and sequential interaction variables were examined for normality and influential outliers. The non-normality of all 4 person-oriented attention variables (infant and maternal overall and average duration) was resolved using square-root transformations. The non-normality of average duration of object-oriented attention for infants and mothers was resolved using natural log transformations and by removing one outlier (> 3.29 *SD* from the mean; Field, 2005) for each variable. The non-normality of three out of four sequential variables could not be resolved without removing outliers. Two outliers were removed from mother responding to infant person-oriented attention, 2 from infant responding to maternal person-oriented attention, and 2 from infant responding to maternal object-oriented attention. Non-normality of all ORs was resolved using natural log transformations. For ease of interpretation, all tables and figures show untransformed data unless otherwise specified.

Birthweight, duration of hospitalization, and corrected age were considered as covariates but were highly related to gestational age and therefore not included in analyses (sharing 72 %, 57 %, and 83 % of variance, respectively). Furthermore, 5-min Apgar scores were skewed and showed limited variability as most infants scored 9 or 10 (out of 10); therefore, it was not possible to include this variable in parametric tests as a covariate. In addition, only 1 out of 12 correlations between Apgar scores and outcome variables was significant (for maternal responding to infant person-oriented attention). Infant gender did not have an effect on infant or maternal person- or object-oriented attention, so all analyses collapsed across infant gender. Gestational age was treated as a continuous variable with a total range of 30–42 weeks, including preterm infants (30–36 weeks) and term infants (37–42 weeks).

### 3.2 Orienting attention to people and objects

To address the first aim, we evaluated whether gestational age was related to the distributions of infant and maternal attention to people versus objects. Table 3 reports descriptive statistics for the overall duration (proportion of interaction) and average duration (per attentional episode) of infant and maternal attention as well as correlations between each of those variables and gestational age. Infants and mothers spent a greater proportion of the interaction attending to objects than to people (for infants 2-tailed *t*(103) = 27.24, *p* < .01, *d* = 4.53; for mothers 2-tailed *t*(103) = 19.16, *p* < .01, *d* = 3.25). Maturity was not related to attention to people: gestational age was unrelated to overall duration or average duration of person-oriented attention for infants and for mothers. Maturity was related to attention to objects. For infants, gestational age was positively related to overall duration and negatively related to average duration of object-oriented attention: less mature infants spent less of the interaction attending to objects, and their attention events lasted longer. For mothers, gestational age was negatively related to overall duration and average duration of object-oriented attention: mothers of less mature infants spent more of the interaction encouraging attention to objects, and their encouraging attention lasted longer.

**Table 3:** Descriptive statistics for infant and mother orienting attention to person versus objects.

		*M*	*SD*	Association with gestational age
**Duration (proportion of interaction)**				
Person-oriented attention	Infant	.09	.09	*r*(102) = −.18, *p* = .065
	Mother	.12	.12	*r*(102) = .01, *p* = .915
Object-oriented attention	Infant	.65	.15	*r*(102) = .24, *p* = .013
	Mother	.58	.16	*r*(102) = −.39, *p* <.001
**Average duration (s)**				
Person-oriented attention	Infant	4.46	3.04	*r*(102) = −.16, *p* = .102
	Mother	11.41	9.92	*r*(102) = −.02, *p* = .813
Object-oriented attention	Infant	13.51	6.47	*r*(101) = −.40, *p* <.001
	Mother	16.65	8.97	*r*(101) = −.46, *p* <.001

*Note.* Untransformed data is presented in this table. However, transformed scores were used in the correlations with gestational age. One outlier was excluded for average duration of maternal object-oriented behavior and one outlier was excluded for average duration of infant object-oriented behavior.

### 3.3 Responding to attention

To address our second aim, we examined infant and maternal responding to partners’ attention across people versus objects. Table 4 reports descriptive statistics for the sequential interaction variables ORs that evaluated contingent responding. *T-*tests were performed separately to determine whether pairs of behaviors were significantly contingent (that is, whether ORs differed significantly from the transformed equivalent of 1; Wickens, 1993). Infant and maternal responding to attention differed according to whether the focus of attention was people versus objects. Infant responding to person-oriented attention, *t*(96) = 9.86, *p* < .001, *d* = 1.00, and maternal responding to person-oriented attention, *t*(92) = 11.53, *p* < .001, *d* = 1.20, were both significantly more contingent than expected by chance (Ferguson, 2009). Infant responding to object-oriented attention was less than expected by chance, *t*(101) = −3.68, *p* <.001, *d* = −0.36. Maternal responding to object-oriented attention was at chance, *t*(102) = 0.13, *p* = .900, *d* = 0.01. We then conducted correlations to examine relations between gestational age and contingent responding to partners’ attention (Table 4). Relations between maturity and responding to attention differed for attention to people versus objects. Gestational age was unrelated to infant responding to mothers’ person-oriented attention but positively related to infant responding to mothers’ object-oriented attention. For mothers, infant gestational age was positively related to responding to infants’ person-oriented attention but unrelated to responding to infants’ object-oriented attention.

**Table 4:** Descriptive statistics for infant and mother responding to attention for person versus object attention.

		*M*	*SD*	Association with gestational age
Responding to Attention (Odds Ratios)				
Person-oriented attention	Infant responding	15.82	46.62	*r*(95) = −.03, *p* = .809
	Mother responding	15.71	36.86	*r*(91) = .21, *p* = .039
Object-oriented attention	Infant responding	0.88	0.54	*r*(100) = .32, *p* = .001
	Mother responding	1.13	0.82	*r*(101) =−.10, *p* = .327

*Note.* ORs represent likelihood of the target behavior occurring within 3 s of the onset of the given behavior than at other times. Untransformed data is presented in this table. However, transformed scores were used in the correlations with gestational age. Data is missing from 9 dyads for mother responding to person-oriented; 5 dyads for infant responding to person-oriented behavior; and 1 dyad for mother responding to object-oriented behavior. Two outliers were removed from mother responding to person-oriented, 2 from infant responding to person-oriented, and 2 from infant responding to object-oriented interactions.

To address our third aim, we examined who leads and who follows in attention sharing when infants are 5 months, and whether leading versus following differs according to infant maturity. In keeping with our overall aims, we examined these relations this separately for person- and object-oriented attention. General linear models (GLMs) were performed with initiator (infant vs. mother) as a within-subjects factor, gestational age as a continuous predictor variable, and the interaction between initiator and gestational age. Overall mothers followed infant attention to both people and objects, but relations between gestational age and following differed between people and objects. For responding to person-oriented attention, there was a main effect of initiator, *F*(1, 90) = 6.00, *p* = .016, *η^2^_p_* = .062, and a significant Initiator x Gestational age interaction, *F*(1, 90) = 6.68, *p* = .011, *η^2^_p_* = .069, but no main effect of gestational age, *F*(1, 90) = 1.43, *p* = .234, *η^2^_p_* = 0.016. The main effect of initiator reflected that mothers responded to their infants’ person-oriented attention more than their infants responded to mothers’ person-oriented attention. The interaction between initiator and gestational age reflected that gestational age was not related to infant responding to mothers’ person-oriented attention (*b* = −0.01, *SE* = 0.04, *p* = .887) but was a significant positive predictor of maternal responding to infants’ person-oriented attention (*b* = 0.08, *SE* = 0.04, *p* = .026). For responding to object-oriented attention, there was a main effect of initiator, *F*(1, 99) = 9.46, *p* = .003, *η^2^_p_* = .087, and a significant Initiator x Gestational age interaction, *F*(1, 99) = 8.50, *p* = .004, *η^2^_p_* = .079, but no main effect of gestational age, *F*(1, 99) = 0.98, *p* = .325, *η^2^_p_* = .010. The main effect of initiator reflected that mothers responded to their infants’ object-oriented attention more than their infants responded to mothers’ object-oriented attention. The interaction between initiator and gestational age reflected that gestational age was positively related to infant responding to mothers’ object-oriented attention (*b* = 0.03, *SE* = 0.01, *p* = .002) but unrelated to maternal responding to infants’ object-oriented attention (*b* = −0.01, *SE* = 0.01, *p* = .203).

## 4 Discussion

The overarching goal of our study was to investigate three factors that might influence attention sharing: infants’ orienting attention to people versus objects, infants’ responding to parental encouragement to attend to people versus objects, and parents’ responding to infant attention to people versus objects. To investigate these relations, we observed 5-month-old term and preterm infants and their mothers during social interactions. We focused on 5 months because we identified it as a transitional age for the development of orienting and responding to attention, and therefore an appropriate age to investigate relations between maturity and attention (Colombo, 2001; Cote & Bornstein, 2018; Perra & Gattis, 2012; Rose et al., 2001; Ross-Sheehy et al., 2017; Tsurumi et al., 2018; van de Weijer-Bergsma et al., 2008; Van Egeren et al., 2001). We separately identified attention to persons and objects based on hypotheses about the specificity of attentional processes, and because both are necessary precursors to sharing attention (e.g., Bakeman & Adamson, 1984; Bornstein et al., 2012; Kaye & Fogel, 1980; Mundy & Newell, 2007). By distinguishing between attention to people and objects, and examining responsiveness to each focus of attention separately, and by comparing attention and responsiveness in healthy term and preterm infants with the same amounts of extrauterine experience but varying gestational ages, we aimed to address and resolve inconsistencies in findings about relations among maturity, attention, and responsiveness.

Our first aim was to evaluate whether maturity affected the distribution of attention to people and objects. Five-month-old infants spent a relatively small proportion of the interaction attending to their social partner, and a substantial proportion of the interaction attending to objects, consistent with previous findings (e.g., Perra & Gattis, 2012). Mothers also spent a small proportion of the interaction encouraging attention to themselves, and a substantial proportion of the interaction encouraging attention to objects. Attention to objects, but not people, was also related to gestational age: more mature infants spent more of the interaction attending to objects, and at the same time their attention to objects occurred in shorter episodes. This finding is consistent with claims that maturity is related to the distribution of attention and to processing speed (Brazelton et al., 1974; Colombo, 2001; Kaye & Fogel, 1980). Maternal attention was related to gestational age in a complementary manner: mothers of more mature infants spent less of the interaction encouraging attention to objects, and those events were briefer.

Our second aim was to evaluate the hypothesis that responding to attention is domain specific rather than general. Infants’ responsiveness differed for attention to people versus objects. Both infants and mothers responded contingently to social attention: When infants attended to their mothers, mothers encouraged attention to themselves, and when mothers encouraged attention to themselves, infants responded accordingly. Neither mothers nor their 5-month-old infants responded contingently to attention to objects. In addition, relations between maturity and responding to attention differed for attention to people versus objects. Infants were less responsive to their mothers’ encouraging attention to objects at younger gestational ages, but their responsiveness to their mothers’ encouraging attention to her did not differ by gestational age. Mothers were less responsive to their infants’ person-oriented attention at younger gestational ages, but their responsiveness to infants’ object-oriented attention did not differ by gestational age. These results are not due to comorbidities and medical complications, which were exclusion criteria for our study. Together the results are consistent with claims that maturity influences the development of disengagement, shifting, and re-engagement of attention as well as processing speed (e.g., Colombo, 2001; Johnson, 1990; Rose et al., 2001). This pattern of responding was however specific to object-oriented attention: Less mature infants were not less responsive to person-oriented attention. In contrast, mothers of less mature infants were less responsive to their infants’ person-oriented attention, but were not less responsive to their infants’ object-oriented attention. Infant and caregiver responsiveness appear to be specific rather than generalize across different foci of attention.

Our third aim was to evaluate who leads and who follows in attention sharing, and whether maturity is related to those roles. Previous evidence indicated that infants become capable of sharing attention with parents and other caregivers toward the end of the first half-year (Mundy & Newell, 2007; Perra & Gattis, 2012). Relatively few studies have examined the question of who leads and who follows to establish attention sharing (Bornstein et al., 2012, 2019; Cote, Bornstein, Haynes, & Bakeman, 2008; Van Egeren et al., 2001), and to our knowledge, no previous study has examined how maturity is related to those roles. As predicted, mothers followed infants’ focus of attention more often than infants followed mothers’ focus of attention. Mothers responded to infant attention to themselves more with increasing gestational age, and infants responded more to mothers’ attention to objects with increasing gestational age. Our findings build on and extend Van Egeren et al.’s (2001) finding that at 4 months infants lead and caregivers follow, but with one important difference. Van Egeren et al. (2001) reported that 4-month-olds followed mothers’ object play more often than mothers followed infants’ object play. Instead, we found that mothers followed their infants’ object-oriented attention more than their infants followed their mothers’ object-oriented attention. Importantly, in our study infants’ following their mothers’ object-oriented attention was positively related to gestational age, suggesting that following object-oriented attention is associated with maturity.

### 4.1 Foundations of attention sharing

Our study addressed a long-standing proposal that a developmental shift in infant attention occurs during the first half year, as infants increasingly attend to objects (Kaye & Fogel, 1980). Our results confirmed that in laboratory-based social interactions 5-month-old infants and their mothers attend more to objects than to people, and importantly, that the distribution of attention is related to maturity. Because we observed parent-infant interactions in a standardized laboratory setting and provided toys, mothers may have interpreted the situation as a tacit instruction to play with objects. Object play, and thus attention to objects, may be less frequent in the home environment. However, the observational setting did not compel mothers to play with objects. In addition, we note that the observational setting and instructions were similar to those used by Perra and Gattis (2012), who observed low levels of object-oriented attention at 2 months, and a significant increase in object-oriented attention from 3 to 4 months, consistent with the levels of object-oriented attention we observed.

Distinguishing between attention to people and objects also allowed us to examine responding to attention to people and objects separately, and thus to address our hypotheses about the heterogeneity of responding to attention. Our fine-grained, microcoding system required categorical and temporal judgments of behavior (Bakeman et al., 2005; Miller, 2011). Agreement on fine-grained, microcoding can be harder to achieve than macrocoding (Miller, 2011), particularly when agreement is evaluated at a second-by-second level, as in this study. Agreement was acceptable but not as high as we would like. Fine-grained, microcoding has many benefits, because of the precision with which hypotheses can be addressed and because it can be combined with sequential analysis and thus strengthens causal inferences (Bakeman & Quera, 2011). Our results confirm and extend previous evidence that parental responding to infant attention is not homogenous, but rather differs according to domain and context (Bornstein & Tamis-LeMonda, 1990; Bornstein et al., 1992, 2008; Bornstein et al., 2012; Vibbert & Bornstein, 1989). Our results also provide evidence that infant responding to parent attention is not homogenous. We found that 5-month-olds respond to person-directed attention, and their responses do not differ according to maturity. By comparison, 5-month-olds did not respond to object-directed attention at the group level, but more mature infants were more likely to do so.

Our results point toward the importance of attention to both people and objects as pre-requisites for joint attention, a foundational process for cognitive and communicative development (Mundy & Newell, 2007; Salley & Colombo, 2016; Salley et al., 2016). Our findings suggest that 5 months is an important transitional period for sharing attention and are consistent with claims that joint attention might emerge from a developmental progression in which infants first share attention with one social partner (dyadic attention) and later share attention with a social partner and some third object (triadic attention) (Kaye & Fogel, 1980; Salley et al., 2016). Our results also indicate that at 5 months, attention sharing is more likely to result from parents’ responding to infants’ attention to objects than infants’ responding to parents’ attention to objects. Furthermore, infants’ responding to parents’ attention to objects increased with maturity. Our cross-sectional (not longitudinal) design did not allow us to observe or evaluate developmental changes at an individual level. Future studies should further evaluate the significance of these early patterns of responding to attention to people versus objects by examining the relations between responding to attention in the first half year with later, more mature forms of initiating and responding in joint attention, and communication more generally (Kuchirko, Tafuro, & Tamis LeMonda, 2018; Landry et al., 1997a; Mundy & Newell, 2007).

### 4.2 Perspectives on preterm birth

To evaluate the role of maturation in orienting and responding to attention, we compared attentional processes in 5-month-old infants born term and preterm. To control for visual and social experience, we observed all infants at the same chronological age, rather than testing infants at corrected ages. We also screened our sample carefully to control for comorbidities and medical complications as well as demographic variables that co-vary with preterm birth. The justification for our study was thus based on the maturation perspective on preterm birth, which assumes that negative outcomes following preterm birth are due to complications, disruptions, or harm to the child, rather than issues inherent to prematurity itself (Bakewell-Sachs, Medoff-Cooper, Escobar, Silber, & Lorch, 2009; Baron, Litman, Ahronovich, & Baker, 2012; Gattis, 2018, 2019). In other words, we treated preterm birth as an “experiment of nature” and used gestational age as a continuous variable allowing us to examine the role of maturity in attentional processes.

Developmental processes are complex, however, and in some cases are highly sensitive to environment and timing. A second perspective on preterm birth, the divergence perspective, assumes that differences in environment and timing due to preterm birth cause the developmental trajectories of preterm children to diverge from those of term children (Aylward, 2005; Gattis, 2018, 2019; van de Weijer-Bergsma et al., 2008). In other words, preterm birth disrupts development, even for children without complications or comorbidities. According to the divergence perspective, preterm birth exerts a persistent influence on development, whether on parent or child, which for some developmental domains become amplified over the course of development, rather than attenuated. The divergence perspective is supported by evidence that some differences between term and preterm children persist beyond corrected ages, and in addition, some differences increase rather than decrease with development (Aylward, 2005; Gattis, 2018, 2019; van de Weijer-Bergsma et al., 2008).

The maturation and divergence perspectives on preterm birth are not mutually exclusive: As would be carried by the Specificity Principle, some developmental outcomes are likely more strongly influenced by maturation whereas other developmental outcomes are likely more strongly influenced by the timing of birth and related environmental inputs (Bornstein, 2017, 2018). Longitudinal evidence is needed to more fully evaluate the consequences of moderate preterm birth for orienting and responding to attention, joint attention, and communicative development during infancy. Future studies should also consider alternative cross-sectional designs. For example, Peña et al. (2014) compared preterm infants with two groups of term infants, one matched on postmenstrual age and one matched on chronological age, and found that the performance of preterm infants on an attention-cuing task was more similar to the term infants who were matched on chronological age and thus had the same amount of extrauterine experience. Such designs minimize the burden of study participation for families of preterm infants and at the same time allow for stronger inferences about the roles of preterm birth, maturation, and experience in development.

Our study addressed inconsistencies in the evidence about how preterm birth is related to orienting and responding to attention in infant-mother dyads by distinguishing between attention to objects and people in a parallel coding system applied to infants and mothers, as well as by treating gestational age as a continuous variable and testing all infants at the same chronological age. Our results indicate that less mature infants engage in fewer, longer bouts of attention to objects compared to more mature infants. Our results also indicate that less mature infants respond less frequently to object-directed attention but do not differ in their response to person-directed attention, whereas mothers of less mature infants respond less frequently to person-directed attention but do not differ in their response to object-directed attention. The juxtaposition of infant and maternal responding suggests the interesting possibility that maternal responses to infant attention reflect an adaptation or accommodation of attentional patterns of preterm infants. Although we are not positioned to draw firm conclusions from this study, future studies should consider detailed longitudinal designs to evaluate this possibility.

Although we compared infants born at different gestational ages to examine the role of maturity in attention, and relied on careful screening of infants to rule out co-morbidities and complications, preterm birth can also influence parents, which may in turn influence their interactions with infants (see Gattis, 2019 for a review). A consistently observed effect is that preterm birth increases stress in parents. We did not evaluate maternal stress and can therefore not rule out the possibility that the mothers of infants born preterm behaved differently in interactions due to stress or other factors besides the relative immaturity of their infants. Future studies of attention sharing in term and preterm infants and their parents should consider measuring parental factors such as stress.

## Data availability statement

Data are available on request from Merideth Gattis, School of Psychology, Cardiff University, Park Place, Cardiff, CF10 3AT. E-mail: GattisM@cardiff.ac.uk.

## Author note

This research was supported by a Waterloo Foundation research project grant (267-1077) awarded to Merideth Gattis, a Wellcome Trust PhD studentship (084911/Z/08/Z) awarded to Alice Winstanley, the Intramural Research program of the NIH/NICHD, USA, and an International Research Fellowship at the Institute for Fiscal Studies (IFS), London, UK, funded by the European Research Council (ERC) under the Horizon 2020 research and innovation programme (grant agreement No 695300-HKADeC-ERC-2015-AdG).

## CRediT authorship contribution statement

**Merideth Gattis:** Conceptualization, Funding acquisition, Investigation, Methodology, Writing - original draft. **Alice Winstanley:** Conceptualization, Formal analysis, Funding acquisition, Methodology, Investigation. **Rebecca Sperotto:** Investigation. **Diane L. Putnick:** Formal analysis, Writing - review & editing. **Marc H. Bornstein:** Conceptualization, Funding acquisition, Methodology, Writing - review & editing.

## Declaration of Competing Interest

The authors declare no conflicts of interest.
